# Supplementary material for: Venomix: a simple bioinformatic pipeline for identifying and characterizing toxin gene candidates from transcriptomic data
Source: PeerJ. 2018 Jul 31;6:e5361. doi: 10.7717/peerj.5361 (PMC6074769; doi:10.7717/peerj.5361)
Supplement: Supplemental Information 6 [file peerj-06-5361-s006.gz › FinalOutput_1E-6/Serine_proteinase-like_BMK-CBP_1/finaltree.pdf]

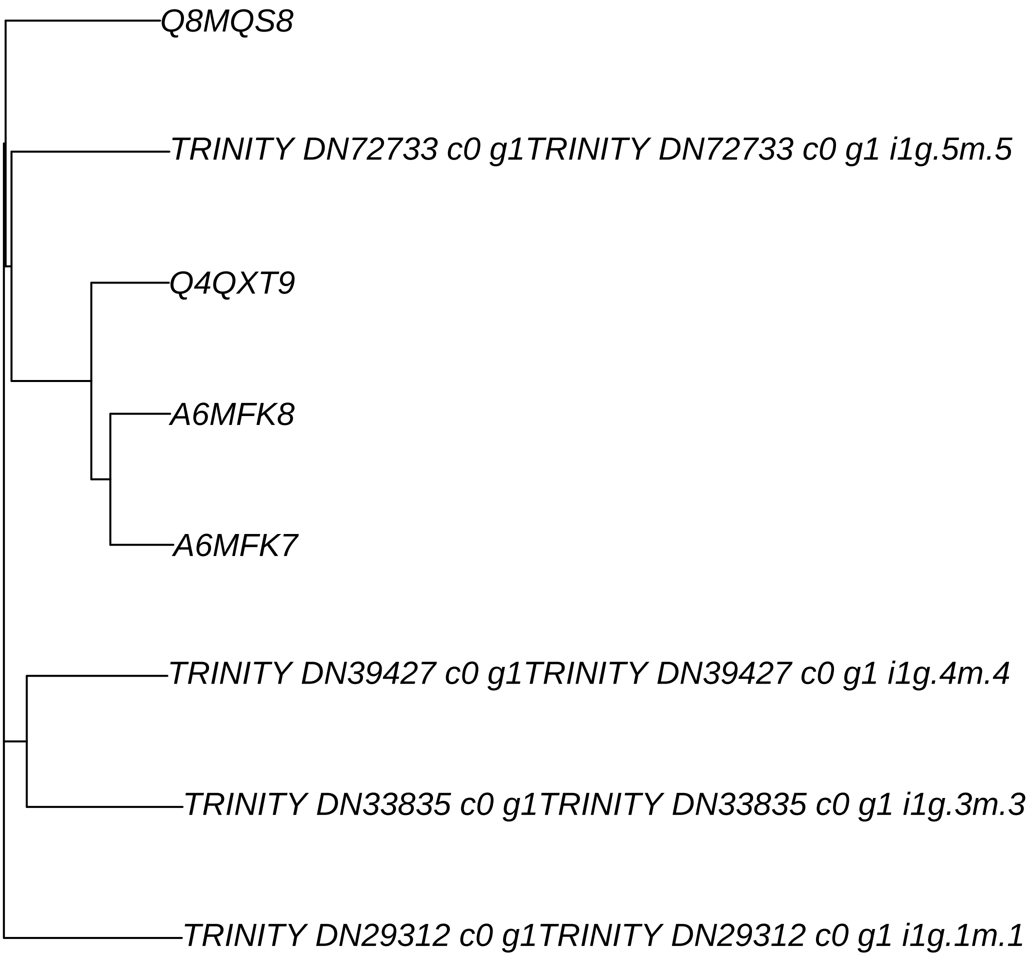
Q8MQS8
TRINITY DN72733 c0 g1TRINITY DN72733 c0 g1 i1g.5m.5
Q4QXT9
A6MFK8
A6MFK7
TRINITY DN39427 c0 g1TRINITY DN39427 c0 g1 i1g.4m.4
TRINITY DN33835 c0 g1TRINITY DN33835 c0 g1 i1g.3m.3
TRINITY DN29312 c0 g1TRINITY DN29312 c0 g1 i1g.1m.1
